# Vadadustat, a HIF Prolyl Hydroxylase Inhibitor, Improves Immunomodulatory Properties of Human Mesenchymal Stromal Cells

**DOI:** 10.3390/cells9112396

**Published:** 2020-11-01

**Authors:** Katarzyna Zielniok, Anna Burdzinska, Beata Kaleta, Radoslaw Zagozdzon, Leszek Paczek

**Affiliations:** 1 Department of Immunology, Transplantology and Internal Diseases, Medical University of Warsaw, Nowogrodzka 59, 02-006 Warsaw, Poland; katarzyna.zielniok@wum.edu.pl (K.Z.); anna.burdzinska@wum.edu.pl (A.B.); radoslaw.zagozdzon@wum.edu.pl (R.Z.); 2 Department of Clinical Immunology, Medical University of Warsaw, Nowogrodzka 59, 02-006 Warsaw, Poland; beata.kaleta@wum.edu.pl; 3 Department of Bioinformatics, Institute of Biochemistry and Biophysics, Polish Academy of Sciences, Pawińskiego 5A, 02-106 Warsaw, Poland

**Keywords:** mesenchymal stem cells, Vadadustat, AKB-6548, preconditioning, priming, immunomodulation, secretome, chemotaxis

## Abstract

The therapeutic potential of mesenchymal stromal cells (MSCs) is largely attributed to their immunomodulatory properties, which can be further improved by hypoxia priming. In this study, we investigated the immunomodulatory properties of MSCs preconditioned with hypoxia-mimetic Vadadustat (AKB-6548, Akebia). Gene expression analysis of immunomodulatory factors was performed by real-time polymerase chain reaction (real-time PCR) on RNA isolated from six human bone-marrow derived MSCs populations preconditioned for 6 h with 40 μM Vadadustat compared to control MSCs. The effect of Vadadustat preconditioning on MSCs secretome was determined using Proteome Profiler and Luminex, while their immunomodulatory activity was assessed by mixed lymphocyte reaction (MLR) and Culturex transwell migration assays. Real-time PCR revealed that Vadadustat downregulated genes related to immune system: *IL24*, *IL1B*, *CXCL8*, *PDCD1LG1*, *PDCD1LG2*, *HIF1A*, *CCL2* and *IL6*, and upregulated *IL17RD*, *CCL28* and *LEP*. Vadadustat caused a marked decrease in the secretion of IL6 (by 51%), HGF (by 47%), CCL7 (MCP3) (by 42%) and CXCL8 (by 40%). Vadadustat potentiated the inhibitory effect of MSCs on the proliferation of alloactivated human peripheral blood mononuclear cells (PBMCs), and reduced monocytes-enriched PBMCs chemotaxis towards the MSCs secretome. Preconditioning with Vadadustat may constitute a valuable approach to improve the therapeutic properties of MSCs.

## 1. Introduction

Human mesenchymal stromal cells (MSCs) therapy has shown a promising potential in the treatment of diseases associated with immune-mediated disorders (reviewed in [1]). Despite the lack of sufficient data explaining the native, physiological function of MSCs in the human body, existing experimental results demonstrate their multipotency [2] and considerable paracrine-mediated immunosuppressive and inflammation resolving activity (reviewed in [3]). As progenitor cells with nonhematopoietic origin, MSCs are isolated from various adult tissues, but the most commonly used source for preclinical and clinical studies is bone marrow, which constitutes the primary niche for this population [4]. Once isolated, MSCs are readily expanded and differentiated in cell culture conditions into several different lineages. Recently, a fundamental shift was made from the initially proposed paradigm of reparative function of MSCs, to the paradigm of MSCs modulating activity, manifested by secretion of numerous bioactive compounds regulating immune response and contributing to tissue regeneration. Encouraging issues favoring the use of MSCs in regenerative therapy are: low immunogenicity, lack of ethical concerns regarding they isolation and use, and an overall minimal risk of their malignant transformation. Moreover, the outstanding potential of MSCs lies in their ability to home to the site of damage and crosstalk with other types of cell in order to limit cell death, diminish an excessive inflammatory response and facilitate the intrinsic tissue regeneration capacity. The therapeutic activity of MSCs seems to be strongly associated with the production of trophic and immunomodulatory factors. A growing number of research indicates, that treatment with MSCs secretome reveals a similar therapeutic effect to MSCs transplantation itself, avoiding the main risks related to allogeneic cell transplantation (reviewed in [5]). Therefore, many attempts have been made to optimize MSCs culture conditions to obtain their most preferred secretory profile. Oxygen tension is one of the major factor closely related to the proliferation, differentiation and stemness of MSCs. However, MSCs are routinely cultured under 21% oxygen pressure conditions that several times exceeds the physiological level available in their natural niches (which ranges from 1% to 7% O_2_) [6,7,8]. There are several reports confirming the beneficial effect of hypoxia and hypoxic preconditioning on migration, regenerative potential, proangiogenic activity and expanded survival of MSCs [9,10,11,12,13,14]. However, culturing cells under low oxygen conditions is demanding, has some limitations and multiplies the costs of MSCs culture. Therefore, the opportunity of using hypoxia mimetic agents for preconditioning of MSCs seems a highly promising approach. The concept is simple. It assumes the use of a drug targeted at cellular hypoxia sensors, which by switching them off triggers cellular response to hypoxia under normoxic conditions. This response is manifested in a number of transcriptional and translational changes leading to regulation of metabolic, proliferation, transport and survival pathways. Detection of oxygen availability occurs in cells mainly through the prolyl-hydroxylase domain family of enzymes (PHDs), which require molecular oxygen to their biological activity. When enough oxygen is present, PHDs are active and hydroxylate specific proline residues (Pro402 and Pro564) in hypoxia inducible factors alpha (HIF-α)—a three isoforms of transcription factor responsible for the expression of hypoxia adaptation genes, of which HIF-1α and HIF-2α are the most important [15]. Hydroxylation of HIF-α proline residues determines its inactivation, being a signal to its ubiquitination and proteasomal degradation. When the oxygen supplies are low, PHDs are inactivated, which results in stabilization of HIF-α and initiation of mechanisms that adapt cells to hypoxia. Several studies have been made to examine the effects of preconditioning MSCs with PHDs inhibitors (reviewed in [16]). To date, various PHDs inactivation strategies have been used in MSCs research (including gene silencing), but only few studies have been performed using selective PHDs inhibitors. Here, we report for the first time how treatment with Vadadustat—a selective HIF PHDs inhibitor—affects paracrine functions and immunomodulatory properties of MSCs. Our findings reveal new aspects of MSCs preconditioning with pharmacologically induced hypoxia, and we strongly believe that may contribute to the improvement of MSCs-based therapies in the treatment of immune disorders.

## 2. Materials and Methods

### 2.1. Isolation and Culture of Human Bone Marrow-Derived Mesenchymal Stromal Cells (BM-MSCs)

BM-MSCs were isolated from bone marrow aspirates of patients without chronic diseases collected during orthopedic surgery (the age and sex profile of donors is provided in Supplementary Table S1). The procedure was performed in accordance with the approval of the Local Bioethics Committee (number KB/115/2016) after receiving informed consent from each patient. Cells were isolated as previously described [17,18]. Briefly, mechanically disassociated bone marrow samples were washed, centrifuged, suspended and seeded on a plastic culture dish (BD Primaria™, BD Biosciences, San Jose, CA, USA) in growth medium composed of low glucose DMEM (Biowest, Riverside, MO, USA) supplemented with 10% FBS (Biowest, Riverside, MO, USA) and antibiotic-antimycotic solution (1% penicillin–streptomycin; 0.5% amphotericin B, Invitrogen, Thermo Fisher Scientific, Waltham, MA, USA). The medium was replaced at day 4, when the first fibroblastic-like colonies of cells were observed do be adhered on a dish. Cells were grown in 5% CO_2_/95% humidified air at 37 °C and the medium was replaced every other day. All experiments were performed on at least 6 individual populations (each population isolated from separate donor), between passage 4–6 and fulfilled currently acknowledged criteria for identification of mesenchymal stromal cells (which we previously described in [19]). 

### 2.2. Human BM-MSCs Identification

#### 2.2.1. Phenotyping of BM-MSCs by Flow Cytometry

BD Stemflow™ hMSC Analysis Kit (BD Biosciences, San Jose, CA, USA) was used to perform BM-MSCs phenotypic characterization. For the purpose of MSCs characterization, cells at passage 4 were stained with antibodies of surface markers CD105 (PerCP-Cy™ 5.5), CD73 (APC), CD90 (FITC) as well as negative expression markers CD45, CD34, CD11b, CD19, HLA-DR (PE) according to the protocol provided by the manufacturer. Flow cytometry analysis was performed on BD FACS Canto II using BD FACS Diva Software (BD Biosciences, San Jose, CA, USA).

#### 2.2.2. Adipogenic Differentiation

To confirm the ability of isolated BM-MSCs to adipogenic differentiation, cells were grown for 3 weeks in differentiating medium consisting of DMEM-high glucose (Biowest, Riverside, MO, USA) supplemented with 10% FBS (Biowest, Riverside, MO, USA) and 1% penicillin–streptomycin (Invitrogen, Thermo Fisher Scientific, Waltham, MA, USA), 10 μg/mL insulin, 60 μM indomethacin, 1 μM dexamethasone and 500 μM 3-isobutyl-1-methylxanthine (IBMX) (all MilliporeSigma, St. Louis, MO, USA). The differentiation medium was replaced every third day. Accumulation of lipid droplets in cells was visualized under a light microscope after the Oil Red O (MilliporeSigma, St. Louis, MO, USA) staining procedure previously described in [19]).

#### 2.2.3. Osteogenic Differentiation

Confirmation of the osteogenic differentiation ability was achieved by culturing cells for three weeks in an osteogenic medium containing DMEM-low glucose (Biowest, Riverside, MO, USA) supplemented with 10% FBS (Biowest, Riverside, MO, USA) and 1% penicillin–streptomycin (Invitrogen, Thermo Fisher Scientific, Waltham, MA, USA), with 100 nM dexamethasone, 10 mM β-glycerophosphate, 50 μM L-ascorbic acid 2-phosphate (all MilliporeSigma, St. Louis, MO, USA). The medium was replaced every third day. Osteogenic differentiation of cells was evaluated by the visualization of calcium deposits by Alizarin Red staining on fixed with 4% paraformaldehyde cells under light microscope. Additionally, the activity of alkaline phosphatase was evaluated using the colorimetric method as previously described in [19].

#### 2.2.4. Chondrogenic Differentiation

Chondrogenic differentiation was achieved in three-dimensional culture of pelleted cells performed in a 15 mL Falcon tube. Chondrogenic medium was composed of DMEM-high glucose (Biowest, Riverside, MO, USA) supplemented with 0.5% FBS (Biowest, Riverside, MO, USA) and 1% penicillin-streptomycin (Invitrogen, Thermo Fisher Scientific, Waltham, MA, USA), with 100 nM dexamethasone, 1% insulin-transferrin-selenium solution (ITS), 10 ng/mL TGFβ2, 100 μM l-ascorbic acid 2-phosphate and 100 μg/mL sodium pyruvate (all MilliporeSigma, St. Louis, MO, USA). 1 × 10^6^ cells suspended in a chondrogenic medium was pelleted by centrifugation and incubated with differentiation medium for three weeks. Until day two, a spheroid cell structure was observed at the bottom of the tube. The medium was changed every third day. The chondrogenic differentiation was conducted for 3 weeks. Then, the microsphere was fixed with 4% paraformaldehyde and underwent a standard histological procedure of paraffinizing, microtome cutting and hematoxilin and eosin as well as Masson trichrome and toluidine blue staining.

### 2.3. Preconditioning of Human BM-MSCs with Vadadustat

In the presented study “pharmacological” hypoxia was achieved by culturing cells with the selective PHDs inhibitor, Vadadustat (AKB-6548, Akebia, Cambridge, MA, USA). Based on preliminary data (Western blot analysis of HIF-1α stabilization and the MTT test) we decided to select the Vadadustat concentration of 40 μM for further studies. Vadadustat was dissolved and stored in −80 °C as 5 mM stock solution in DMSO according to the manufacturer instruction. Notably, no more than 0.8% (*v*/*v*) of DMSO was finally present in the culture medium, which did not cause any noticeable cytotoxic effect (MTT analysis presented in Supplementary Figure S1). The control group of MSCs was incubated with the same dose (0.8% *v*/*v*) of DMSO alone.

### 2.4. BM-MSCs RNA Isolation

For isolation of RNA, cells from 6 donors were cultured in 60 mm dishes until approximately 70% confluency was reached. MSCs were then exposed to experimental conditions. Both, control cells and 40 μM Vadadustat-treated cells were incubated at an atmospheric O_2_ concentration. After 6 h treatment all cells were washed and disrupted in 350 μL of RLT buffer from the Qiagen RNeasy Mini Kit (Qiagen, Hilden, Germany). Samples were then stored in −80 °C until further use. RNeasy Mini Kit was used for total RNA extraction from MSCs according to the manufacturer’s isolation protocol. The concentration and integrity of collected RNA samples were determined spectrophotometrically using NanoDrop 1000 (NanoDrop Technologies, Thermo Fischer Scientific, Waltham, MA, USA) and Bioanalyzer Chip RNA 7500 series II (Agilent Technologies, Santa Clara, CA, USA).

### 2.5. Gene Expression Analysis by Real-Time PCR

Reverse transcription was performed using a High Capacity cDNA Reverse Transcription Kit (Applied Biosystems, Thermo Fisher Scientific, Waltham, MA, USA). A quantity of 2 μg of total RNA was converted to cDNA according to producer’s instruction. Real-time PCR was conducted using SYBR Select Master Mix (Applied Biosystems, Thermo Fisher Scientific, Waltham, MA, USA) in a 7500 Real-Time PCR System (Applied Biosystems, Thermo Fisher Scientific, Waltham, MA, USA). In each 20 μL reaction 100 ng of cDNA template and 0.5 μM forward and reverse primers was used. PCR reaction was started with two initial steps at 50 °C and 95 °C each for 2 min, followed by 40 cycles of 95 °C for 15 s and 60 °C for 1 min respectively. Standard curves were run on each plate to determine the amplification efficiency. Primer pairs were purchased from the Laboratory of DNA Sequencing and Oligonucleotide Synthesis, Institute of Biochemistry and Biophysics (IBB), Polish Academy of Sciences, Warsaw, Poland (oligo.pl) and -MilliporeSigma, St. Louis, MO, USA). Primer pairs sequences of examined genes are: *IL1B* FRD: 5′-CCACAGACCTTCCAGGAGAATG-3′, REV: 5′-GTGCAGTTCAGTGATCGTACAGG -3′; *IL24* FRD: 5′-CTTCTCTGGAGCCAGGTATCAG-3′, REV: 5′-GGCACTCGTGATGTTATCCTGAG-3′; *CCL28* FRD: 5′-CTGGAAAGAGTGAATATGTGTC -3′, REV: 5′-CTTGACATGAAGGATGACAG-3′; *ICAM1* FRD: 5′-ACCATCTACAGCTTTCCG-3′, REV: 5′-TCACACTTCACTGTCACC-3′; *IL1R1* FRD: 5′-ATTTAAGCAGAAACTACCCG-3′, REV: 5′-TTGCAATCCTTATACCACTG-3′; *LIFR* FRD: 5′-AAGTTTATCCCCATACTCCTAC-3′, REV: 5′-CCTGGTAAATGCCAAGAAAG-3′; *HIF1A* FRD: 5′-GAAACTACTAGTGCCACATC-3′, REV: 5′-GGAACTGTAGTTCTTTGACTC-3′; *IL6* FRD: 5′-GCAGAAAAAGGCAAAGAATC-3′, REV: 5′-CTACATTTGCCGAAGAGC-3′; *CCL2* FRD: 5′-AGACTAACCCAGAAACATCC-3′, REV: 5′-ATTGATTGCATCTGGCTG-3′; *TGFB3* FRD: 5′-TGTTGAGAAGAGAGTCCAAC-3′, REV: 5′- ATCACCTCGTGAATGTTTTC-3′; *IL23A* FRD: 5′-AGATAAATCTACCACCCCAG-3′, REV: 5′-CACATGTCAGTCAGTATTGG-3′; *CXCL8* FRD: 5′-GTTTTTGAAGAGGGCTGAG-3′, REV: 5′-TTTGCTTGAAGTTTCACTGG-3′; *IL17RD* FRD: 5′-AGTAGCTTCAAAAGAACTGG-3′, REV: 5′-CTCGGGTTCTAAAGAAGAAAG-3′; *PDCD1LG1* FRD: 5′-GGCATCCAAGATACAAACTCAA -3′, REV: 5′-CAGAAGTTCCAATGCTGGATTA-3′; *PDCD1LG2* FRD: 5′-GAGCTGTGGCAAGTCCTCAT-3′, REV: 5′-GCAATTCCAGGCTCAACATTA-3′; *B2M* FRD: 5′-TGGAGGCTATCCAGCGTACT-3′, REV: 5′-CGGATGGATGAAACCCAGAC-3′. Primer pairs for *LEP* (Cat. no. qHsaCID0017538) and *TNF* (Cat. no. qHsaCED0037461) were purchased from Bio-Rad Laboratories, Inc (Hercules, CA, USA). The relative quantification of a fold change in gene expression was calculated using the Pffafl method based on ΔCt and amplification efficiency of the transcripts normalized to the B2M (β2-microglobulin) reference gene [20]. The expression of each gene in control samples was appointed as 1. The analysis was performed in triplicate on cell populations from at least 6 BM-MSCs donors.

### 2.6. Analysis of BM-MSCs Cytokine Secretion by Antibody Array Proteome Profiler

The relative changes in secretory activity of Vadadustat treated BM-MSCs compared to control cells were examined using the Proteome Profiler Human XL Cytokine Array (Cat. no. ARY022B, R&D Systems, Bio-Techne, Minneapolis, MN, USA). The Proteome Profiler membrane-based antibody array enables to simultaneously measure the relative level of 102 human cytokines in a single sample. For the purpose of this assay, BM-MSCs from 6 donors were grown in a standard growth medium on a 6-well plates until approximately 80% confluency was achieved. 24 h before the start of the experiment, all cells were primed with IFNγ (25 ng/mL, MilliporeSigma, St. Louis, MO, USA). Next day, cells were washed and culture medium was replaced with OptiMEM Medium, no phenol red (Gibco, Thermo Fischer Scientific, Waltham, MA, USA) with reduced FBS content to 4% and supplemented with 1.0% penicillin–streptomycin with/without Vadadustat 40 μM. Cells of each population were treated in triplicate. After 24 h treatment cells supernatants were collected in an Eppendorf tube (1.5 mL), centrifuged at 4500 rpm for 5 min, transferred to new tubes, mixed and divided into 200 µL aliquots and frozen in −80 °C. Prior to the analysis, cell supernatants from 6 donors were thawed on ice and pooled. The analysis was performed according to the manufacturer’s instruction. Chemiluminescence of membranes was detected with ChemiDoc MP Imaging System (Bio-Rad Laboratories Inc., Hercules, CA, USA) and the integrated optical density of each spot was measured and quantified using Image Lab software (Bio-Rad Laboratories Inc., Hercules, CA, USA).

### 2.7. Quantitative Analysis of BM-MSCs Cytokine Secretion by Luminex Multiplex Immunoassay

The quantitative analysis of selected cytokines by the Luminex method was performed on cell supernatants, the preparation of which was described above. Samples were not pooled in this analysis, so supernatants from six populations were analyzed separately. The custom Luminex Multiplex kit was purchased in R&D Systems (Bio-Techne, Minneapolis, MN, USA) and contained IL6, CXCL8, IL4, IL10 and HGF analytes. The procedure was performed according to the manufacturer’s instructions. The flow based magnetic beads reading was performed on Luminex LX-200 Instrument (Thermo Fisher Scientific, Waltham, MA, USA). All samples were analyzed in duplicate.

### 2.8. Isolation and Identification of Peripheral Blood Mononuclear Cells (PBMCs)

Human PMBCs used in this study were freshly isolated from buffy coats each time. Buffy coats were purchased at the Regional Blood Donation and Blood Treatment Centre in Warsaw as medical waste from whole blood, which was centrifuged without a density gradient. The isolation of PBMCs was performed within 4 h of collecting whole blood. Buffy coats were first diluted in PBS (without calcium and magnesium) in 50 mL Falcon tubes, and then cells were separated by density gradient centrifugation on Histopaque-1077 (MilliporeSigma, St. Louis, MO, USA). PBMCs were collected from a plasma/Ficoll interface with a Pasteur pipette and transferred to a new 50 mL falcon tube. Isolated PBMCs were then washed four times in PBS to rinse cells pellets and to reduce platelet contamination. Finally, cells were suspended in growth medium composed of RPMI-1640 (Thermo Fischer Scientific, Waltham, MA, USA) with 10% human serum (Biowest, Riverside, MO, USA) and 1% penicillin-streptomycin solution (Invitrogen, Thermo Fisher Scientific, Waltham, MA, USA) and counted. To obtain the monocyte-enriched population used in the migration assay, isolated PBMCs were seeded on Primaria™ Tissue Culture Dishes at a density of 75 × 10^4^/mL in RPMI-1640 (Biowest, Riverside, MO, USA) without serum. After two hours, the plates were vigorously washed five times with PBS, then the adherent cells remaining on the dishes were scraped off and suspended for further processing. The flow cytometric analyses of the PBMCs and monocyte-enriched were performed by CD3 (PerCP Mouse anti Human Clone SP34-2), CD14 (FITC Mouse Anti-Human Clone M5E2) and CD16 (PE-Cy™7 Mouse Anti-Human Clone 3G8) staining (all BD Biosciences, San Jose, CA, USA) on BD FACS Canto II using BD FACS Diva Software (BD Biosciences, San Jose, CA, USA) and analyzed by FCSExpress7 (De Novo Software, Glendale, CA, USA).

### 2.9. Mixed Lymphocyte Reaction (MLR) Assay

For the purpose of MLR assay human PBMCs were isolated from buffy coats from 6 healthy blood donors. The assay was performed in three independent sets of experiments on two donors each. Supernatants from 6 populations of IFNγ (25 ng/mL) primed BM-MSCs treated for 24 h with/without Vadadustat 40 μM were used to determine the effect of Vadadustat pretreatment on immunomodulatory activity of MSCs secretome. In this study, half of the isolated PBMCs were inactivated for 90 min with γ-irradiation. Next, 1 × 10^5^ both responder (active) and irradiated (stimulatory) PBMCs were seeded into wells of 96-well plates in a combination of auto- (AA_ir_, BB_ir)_ and allo- (AB_ir_, BA_ir_) stimulation. Cells were maintained in RPMI-1640 (Thermo Fischer Scientific, Waltham, MA, USA) supplemented with 10% FBS (Gibco, Thermo Fisher Scientific, Waltham, MA) and antibiotic–antimycotic solution (1% penicillin-streptomycin; 0.5% amphotericin B, Invitrogen, Thermo Fisher Scientific, Waltham, MA). The MLR assay were performed using 96-well plates. In the part of the wells where the direct effect of Vadadustat on auto- and allostimulated PBMCs as well as its effect on the interaction between MSCs and PBMCs were studied, 40 μM Vadadustat was added to the experimental wells daily as a stock solution. Control wells were treated daily with equivalent volumes of DMSO. In the remaining wells, in which the indirect effect of Vadadustat pre-conditioning on the interaction between MSCs and PBMCs was studied, a 1:1 mixture of RPMI-1640 growth medium and supernatants from 24 h cell culture of control or Vadadustat preconditioned MSCs was added once at the beginning of the experiment. Plates were then cultured for 5 days at 37 °C in a humidified atmosphere with 5% CO_2_. After 5 days of cell culture, PBMCs were pulsed with 1 μCi/well of 3H-thymidine (113 Ci/nmol, NEN) for the last 18 h of incubation and then harvested with an automated cell harvester (Skatron). The 3H-thymidine incorporation into cells was measured based on the level of radioactivity reported as ‘Corrected Counts per Minute’ (CCPM) using a scintillation counter (Wallac, PerkinElmer, Inc., Waltham, MA, USA). All treatments were performed in triplicate.

### 2.10. Transwell Migration Assay

The effect of 40 μM Vadadustat preconditioning on the chemotactic properties of the BM-MSCs secretome was investigated using a “96 Well Cell Migration Assay” reagent kit from Cultrex^®^ (cat. no. 3465-096-K) (R&D Systems, Bio-Techne, Minneapolis, MN, USA), which utilize a simplified design of a Boyden chamber with polyethylene terephthalate (PET) membrane with pores of 8 μm size. For the migration test, we used monocyte-enriched PBMCs (*n* = 4) suspended in RPMI (Biowest, Riverside, MO, USA) containing 0.5% human serum (Biowest, Riverside, MO, USA) at a density of 4 × 10^6^/mL. 50 μL of cell suspension from each donor were applied to the upper chambers of the plate (2 × 10^4^ cells per well), each in duplicate. Quantities of 150 µL per well of growth medium (RPMI with 0.5% human serum) or freshly thawed, pooled supernatants from cultures of 7 MSCs populations were applied to the bottom chambers of the plate. Each of three treatments: growth medium alone, supernatants from control MSCs and MSCs preconditioned with Vadadustat was applied in duplicate. Plates were then incubated under standard conditions (37 °C, 5% CO_2_) for 48 h. After incubation, the upper chambers were carefully aspirated and the cells that migrated to the bottom compartments of the plate were detached using a cell dissociation solution with calcein acetomethylester (calcein-AM). Afterwards, plates were incubated at 37 °C for 30 min. During this time, cells internalized calcein-AM, and cellular esterases then cleaved it into free calcein. Released calcein possess strong fluorescence, that was used to estimate the number of migrated cells. After incubation, plates were disassembled and bottom chambers were fluorescently read at 485 nm excitation and 520 nm emission on Perkin Elmer Victor X4 plate reader (PerkinElmer, Inc., Waltham, MA, USA). The degree of cell migration was assessed by comparing fluorescence in the wells with MSCs culture supernatants to fluorescence in wells with growth medium alone, and expressed as the ratio of migrating cells.

### 2.11. Statistical Analysis

The results were statistically analyzed using STATISTICA 13.1 software (Tibco, Palo Alto, CA, USA). Shapiro-Wilk test was used to analyze the data distribution within groups. Wilcoxon matched-pairs signed-rank test was used to determine statistical significance between two groups of related data with abnormal distribution. Student’s t-test was used to evaluate significance between two groups of related data with confirmed normal distribution. A *p*-value of < 0.05 (*) was considered statistically significant, and *p* < 0.01 (**), or *p* < 0.001 (***) as highly significant. Graphs are presented as mean ± SEM (standard error of the mean) unless otherwise indicated.

## 3. Results

### 3.1. Isolation and Characterization of Human BM-MSCs

**Figure 1:**
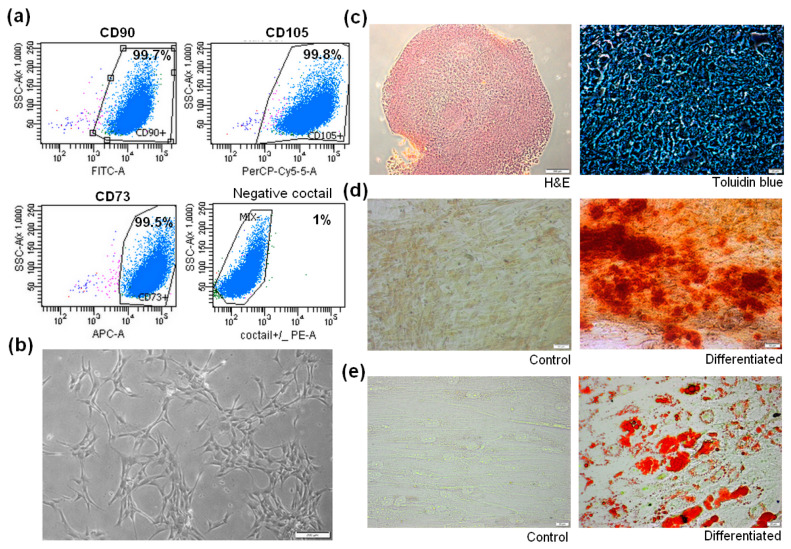
Identification and morphology of human bone marrow-derived mesenchymal stromal cells (hBM-MSCs). (**a**) Flow cytometry analysis of representative MSCs population. MSCs were positive for CD73, CD90, CD105 and negative for CD34, CD45, CD11b, CD19, HLA-DR. (**b**) Representative image of undifferentiated BM-MSCs morphology cultured under standard growth condition (21% O_2_ and 5% CO_2_). Light microscopy, image scale 200 μm (**c**) chondrogenic differentiation of BM-MSCs. Hematoxylin-eosin (HE) and Toluidine blue staining of BM-MSCs microsphere section. Light microscopy, HE staining scale 200 μm, Toluidine blue staining scale 20 μm. (**d**) Osteogenic differentiation of BM-MSCs. Control and differentiated cells stained with Alizarin red. Light microscopy, scale 50 μm. (**e**) Adipogenic differentiation of BM-MSCs. Oil Red O staining of control and differentiated cells. Light microscopy, scale 20 μm.

MSCs isolated from bone marrow were identified according to the International Society for Cell and Gene Therapy (ISCT) statement established in 2006 [21]. All isolated cell populations were proven to form colonies and adhere to a plastic culture surface (a representative population is shown in Figure 1b). The mean expression of surface markers from the 7 BM-MSCs population was: CD73—99.5%, CD90—98.8%, CD105—99.4%, and no antigens CD45, CD34, CD11b, CD19 and HLA-DR were detected on 97.6% cells (Supplementary Table S2). Figure 1a shows a representative panel of BM-MSCs phenotyping results using flow cytometry (full panel in Supplementary Table S3). Tri-lineage differentiation capability was confirmed as shown in Figure 1c–e.

### 3.2. Vadadustat Preconditioning of BM-MSCs Affected the Expression of Genes Associated with the Regulation of Immune Responses

**Figure 2:**
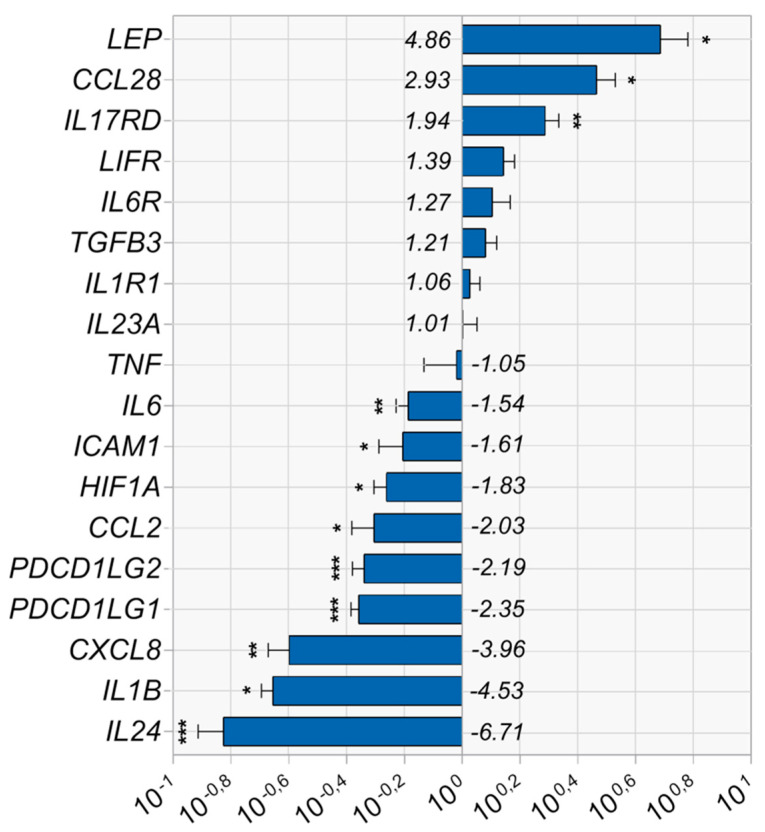
Relative expression of chosen immune system-related genes performed by real-time PCR on 6 bone marrow-derived mesenchymal stromal cells (BM-MSCs) populations incubated for 6 h with Vadadustat or under standard culture conditions (control). Results are presented as fold change in gene expression in MSCs cultured with Vadadustat versus control MSCs ± SEM (standard error of the mean), calculated by the Pfaffl method [20]. * indicates the statistically significant (*p* < 0.05), ** (*p* < 0.01) and *** (*p* < 0.001) highly statistically significant differences obtained by Student’s paired t-test (for data with normal distribution) or Wilcoxon matched-pairs signed rank test (for data with abnormal distribution) of ΔCt values of Vadadustat-treated samples in relation to ΔCt of control samples.

Due to our particular interest in the regulation of immune functions by MSCs, we analyzed the expression of genes related to their immunomodulatory properties. The list of examined genes was based on our previous study evaluating Vadadustat-induced changes in the MSCs transcriptome obtained by RNA sequencing (currently under review). The list included genes encoding factors secreted by MSCs in response to immune stimuli (CCL2, IL6, CXCL8 and TNF), proteins involved in signaling pathways activated by cytokines (e.g., IL17RD, LIFR, IL6R and ICAM1) and other immune regulatory molecules PDCD1L1, PDCD1L2 or LEP (genes listed in Figure 2). We performed real-time PCR analysis of selected genes on the 6 BM-MSCs populations incubated for 6 h under standard grown conditions (control) or with 40 μM Vadadustat (Figure 2).

The results clearly demonstrate, that Vadadustat strongly down-regulated the expression of *IL24* (−6.71), *IL1B* (−4.53), *CXCL8* (−3.96) and slightly *PDCD1LG1* (−2.35), *PDCD1LG2* (−2.19), *CCL2* (−2.03), *HIF1A* (−1.83), *ICAM1* (−1.61) and *IL6* (−1.54), and up-regulated the expression of *IL17RD* (1.94), *CCL28* (2.93) and *LEP* (4.86) compared with control cells. The results obtain for *TNF* (−1.05), *IL23A* (1.01), *IL1R1* (1.06), *TGFB3* (1.21), *IL6R* (1.27), *LIFR* (1.39) did not differ significantly between control cells and treated with Vadadustat.

### 3.3. Functional Activity of BM-MSCs Preconditioned with 40 μM Vadadustat

Genes expression analysis of Vadadustat-preconditioned BM-MSCs showed promising results due to its potential influence on MSCs immunomodulatory properties. At the next stage, it was crucial to determine whether these changes are reflected in the functional activity of MSCs. Several analyzes related to MSCs activity were performed to determine whether this method of MSCs preconditioning could enhance their immunosuppressive potential.

#### 3.3.1. Preconditioning with Vadadustat Changed the Secretory Profile of BM-MSCs

**Figure 3:**
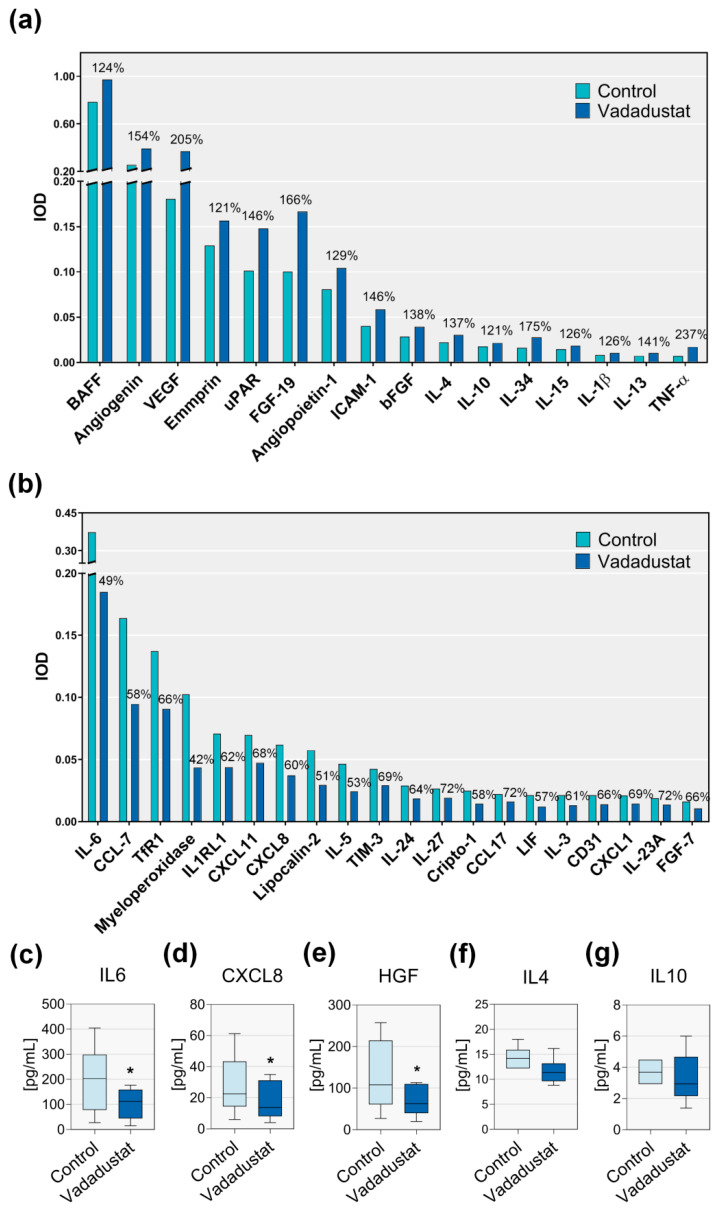
Analysis of the secretome from 6 populations of human bone marrow-derived mesenchymal stromal cells (hBM-MSCs) pretreated with Vadadustat for 24 h. (**a**,**b**) Proteome profiler analysis of cytokines and chemokines whose secretion by MSCs: (**a**) increased or (**b**) decreased by at least 20% as a result of 24 h pretreatment with 40 μM Vadadustat. Bars represent IOD (integrated optical density) of antibodies-dots measured by chemiluminescent detection. The values above the bars show what percentage of control MSCs secretion (indicated as 100%) are values obtained after incubation with Vadadustat. Samples from 6 MSCs populations were pooled for the analysis. (**c**–**g**) Quantitative evaluation of (**c**) IL6, (**d**) CXCL8, (**e**) HGF, (**f**) IL4 and (**g**) IL10 level in the secretome of 6 BM-MSCs populations cultured under control conditions or preconditioned with Vadadustat, conducted using Luminex assay. The samples were not pooled for analysis. Boxes show quartiles of secreted cytokine amount in pg/mL with median, whiskers represent “min to max” values. * indicates the statistically significant difference (*p* < 0.05) by Wilcoxon matched-pairs signed-rank test in the groups of related data with abnormal distribution, and by Student’s t-test in the groups of related data with confirmed normal distribution.

The effect of Vadadustat preconditioning on BM-MSCs cytokine and chemokine secretion profile was determined after 24 h cells treatment with 40 μM Vadadustat by the antibody based Proteome profiler array (Figure 3a,b).

The analysis of cytokines and chemokines found in the pooled supernatants of 6 BM-MSCs populations treated with Vadadustat showed a number of factors whose secretion changed when compared to the control cell supernatants. The observed changes concerned both compounds whose secretion increased (Figure 3a) as well as those whose secretion decreased (Figure 3b). Analysis of the results indicated that among compounds whose secretion was downregulated by Vadadustat treatment, the most prominent decrease was observed among the cytokines secreted in large quantities by MSCs. The marked decrease in secretion was noted for myeloperoxidase (to 42% of the control value), IL6 (to 49%) and CCL7 (to 58%). A decrease in secretion was also noted among compounds secreted by MSCs in smaller amounts: lipocalin 2 to 51%, IL5 to 53%, LIF to 57%, Cripto1 to 59%, CXCL8 to 60%, IL3 to 61% and IL24 to 64%. In addition, the level of five flagship cytokines related to MSC immunomodulation: IL6, CXCL8, HGF, IL4, IL10 was measured in MSCs secretome using the Luminex method. MSCs preconditioning with Vadadustat resulted in a reduction of IL6 secretion from an average of 199 pg/mL to 103 pg/mL (49% decrease, Figure 3c). The CXCL8 secretion decreased on average from 24.59 pg/mL in control cells to 15.46 pg/mL in those treated with Vadadustat (Figure 3d). HGF secretion was reduced from an average of 128 pg/mL to 68 pg/mL (47% decrease, Figure 3e). There were no statistically significant changes in the secretion of IL4 (14 ng/mL vs. 11 pg/mL, Figure 3f) and IL10 (3.7 pg/mL vs. 3.1 pg/mL, Figure 3g) related to Vadadustat pretreatment.

#### 3.3.2. Vadadustat Significantly Increased the Inhibitory Effect of MSCs on Proliferation of Allostimulated PBMCs

**Figure 4:**
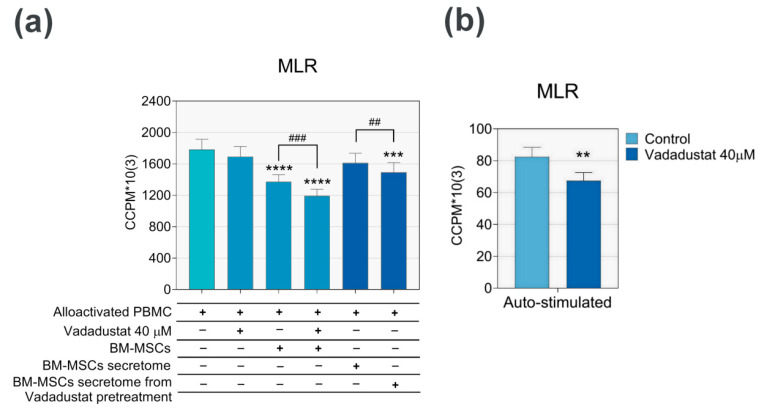
Mixed lymphocyte reaction (MLR) assay. The effect of 40 μM Vadadustat on the allo- and auto-responsiveness of leukocytes. (**a**,**b**) Assessment of peripheral blood mononuclear cells (PBMCs) activation and proliferation capacity in response to direct and indirect effects of Vadadustat by MLR. MLR was performed on freshly isolated PBMCs from 6 donors, 6 populations of bone marrow-derived mesenchymal stromal cells (BM-MSCs) and supernatants pooled from the culture of 6 BM-MSCs populations. The experiment was conducted for 5 days. Results are presented as: (**a**) Effect of Vadadustat on PBMCs alloreactivity and PBMCs–MSCs interaction, and (**b**) effect of Vadadustat on PBMCs autoreactivity. Both assessed by measuring radioactivity of 3H-thymidine-incorporated cells and reported as mean ‘Corrected Counts per Minute’ (CCPM) ± SEM (standard error of the mean). ** indicates the statistically significant difference for *p* < 0.01, *** for *p* < 0.001, **** for *p* < 0.0001 by Friedman test with Dunn’s multiple comparison of mean rank of each group with a mean rank of an alloactivated PBMC (control) in the groups of related data with abnormal distribution. ## indicates statistically significant (*p* < 0.01) and ### (*p* < 0.001) highly significant differences between the two treatment groups analyzed by Wilcoxon matched-pairs signed-rank test in the groups of related data with abnormal distribution, and by Student’s t-test in the groups of related data with confirmed normal distribution.

A series of MLR assays were performed to evaluate the effect of Vadadustat preconditioning on the inhibitory properties of BM-MSCs on PBMCs. The assays examined both the direct effect of Vadadustat on the PBMCs and MSCs–PBMCs interaction as well as the indirect effect of incubating PBMCs with supernatants from Vadadustat-preconditioned MSCs. PBMCs were obtained from 6 donors and 6 BM-MSC populations were used. The results presented in Figure 4a clearly demonstrate that Vadadustat enhanced an immunosuppressive activity of MSCs on PBMCs.

The PBMCs co-culture with control MSCs resulted in a statistically highly significant 22% decrease in PBMCs proliferation compared to the value of allostimulated PBMCs (control). Furthermore, five-day treatment of PBMCs-MSCs culture with Vadadustat resulted in a greater, 28% decrease in allostimulated PBMCs proliferation compared to control cells. When the same PBMCs were cultured for 5 days with the MSCs secretome (1:1 mixture of MSCs supernatants and RPMI growth medium), a significant inhibition of PBMCs proliferation was also noted. The secretome of control MSCs caused a decrease in PBMCs alloreactivity by 9%, and of MSCs preconditioned with Vadadustat by 16% compared to allostimulated PBMCs. However, in both direct and indirect Vadadustat treatments, a similar percentage decrease in PBMCs proliferation between the MSCs alone and preconditioned with Vadadustat was determined (6–7%). In both treatments, the effect of Vadadustat was statistically highly significant. Due to the very short half-life of Vadadustat (4.5 h according to the manufacturer [22]) in the collected secretome of Vadadustat preconditioned MSCs (24 h incubation) there should no longer be an active inhibitor. We noted lack of HIF-1a stabilization after 24 h MSCs incubation with Vadadustat, confirmed by Western blot analysis (data not shown). Thus, we argue that the observed suppressive effect of secretome from Vadadustat preconditioned MSCs was related only to the secretory activity of cells and not to Vadadustat itself. However, in some part Vadadustat possess the direct effect on PBMCs proliferation as well. Treatment of allostimulated PBMCs with Vadadustat caused a 5% decrease in their proliferation. A much larger decrease in proliferation resulting from Vadadustat was noted in autostimulated PBMCs (Figure 4b), which reached 18%. However, it should be noted that PBMCs autoresponsiveness remained at a much lower level. To summarize, the observed suppressive effect of Vadadustat on the reactivity of PBMCs was both, associated with the changes in MSCs activity as well as the direct effect on PBMCs.

#### 3.3.3. Secretome from Vadadustat Preconditioned MSCs Significantly Reduced PBMCs Migration

**Figure 5:**
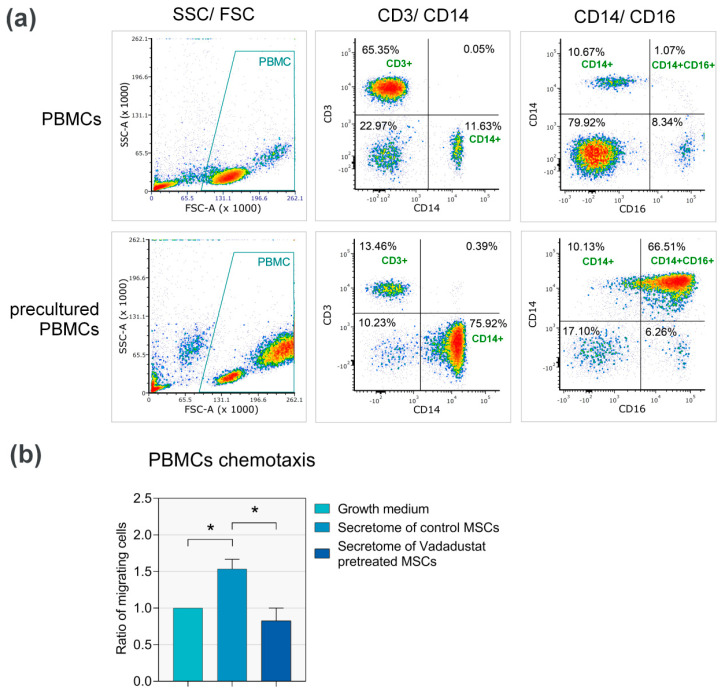
Assessment of the chemotactic properties of the secretome of Vadadustat preconditioned bone marrow-derived mesenchymal stromal cells (BM-MSCs). (**a**) Flow cytometric analysis of lymphocyte populations in peripheral blood mononuclear cells (PBMCs) isolated or precultured in plates based on CD3 (PerCP), CD14 (FITC) and CD16 (PE-Cy7) staining. PBMCs were gated based on FSC and SSC, percentages of monocytes (CD14^+^) and activated monocytes (CD14^+^CD16^+^) in PBMCs are shown in quadrants. (**b**) The analysis of chemotactic properties of secretome from 6 BM-MSCs populations cultured for 24 h under standard conditions (control) and with Vadadustat on the migration of monocyte-enriched peripheral blood mononuclear cells (PBMCs) (precultured PBMCs). The rate of cell migration is presented as the ratio of migrating cells ± SEM (standard error of the mean), obtained by comparing the fluorescence of PBMCs migrated to the growth medium (denoted as 1) with the fluorescence of PBMCs migrated into the secretome of control and Vadadustat pretreated MSCs. * Indicates the statistically significant (*p* < 0.05) differences obtain by Wilcoxon matched-pairs signed-rank test in the groups of related data with abnormal distribution, and by Student’s t-test in the groups of related data with confirmed normal distribution.

96 Well Cell Migration Assay was performed to assess the effect of Vadadustat preconditioning on the chemotactic properties of BM-MSCs secretome. We quantified the degree of 5 donors’ PBMCs migration through a 8 micron PET membrane in response to stimulating and/or inhibiting compounds contained in pooled supernatants collected from 7 MSCs populations incubated for 24 h with Vadadustat, under standard growth conditions or with growth medium alone. For the purpose of migration analysis, we used PBMCs fraction with enriched monocyte content. We obtained the monocyte-enriched PBMCs by pre-culturing the cells on plates for 2 h, and applying a 5× PBS wash to leave only adherent cells. Phenotypic analysis of precultured PBMCs by flow cytometry showed that nearly 76% of the cells used in the assay were monocytes (CD14^+^), of which 66.5% were activated monocytes (CD14^+^CD16^+^) (Figure 5a).

Cell migration from the inserts to the basal compartment of plate wells was assessed after 48 h of monocyte-enriched PBMCs incubation. The results presented in Figure 5b show that within 48 h of incubation there was a significant, 53% increase in PBMCs/monocytes migration in wells containing secretome of control MSCs compared to wells with growth medium alone. Conversely, there was a statistically significant 46% decrease in cell migration in wells with secretome of Vadadustat-preconditioned MSCs compared to migration in wells with secretome of control MSCs. Moreover, there was a slight (17%) decrease in cell migration by comparing Vadadustat supernatants with the growth medium alone, although this effect was not statistically significant.

## 4. Discussion

In recent years, great efforts have been made to develop methods for obtaining more effective and safer MSCs for use in cell therapy. Many studies were carried out to determine the role of MSCs in the regulation of the immune system, showing that their immunomodulatory capacity is a very plastic feature [23]. The plasticity of MSCs immunomodulation is associated with the ability to elicit markedly different modulatory responses, which results from the current state of inflammatory mediators in their microenvironment. Development of a chronic inflammatory microenvironment, resulted from loss of peripheral immune tolerance and excessive stimulation of innate and adaptive immune responses, is associated with the course of autoimmune diseases. MSCs can target such an inflammatory microenvironment by paracrine actions, demonstrating broad immunosuppressive, anti-fibrogenic, anti-apoptotic and pro-angiogenic effects [24]. Immunomodulation attributed to the therapeutic activity of MSCs is related to their function to modulate the proliferation, differentiation, adhesion, and migration of immune cells under disease conditions. Since the immunosuppressive nature of MSCs activity is generally therapeutically desirable, many approaches have been developed to modulate the culture conditions of MSCs in order to obtain their inflammatory-resolving phenotype. While a number of MSCs preconditioning strategies are currently being investigated, cytokine priming and recently hypoxic pretreatment appear to be the major approaches used to increase MSCs immunomodulatory properties [25,26]. Moreover, recent findings indicate that hypoxia inducible factor-1α (HIF-1α) is a major regulator of the immunomodulatory functions of MSCs [27,28]. Although the effect of HIF-1α stabilization by hypoxia mimetic agents on MSCs properties has already been studied (cobalt chloride, deferoxamine, ciclopirox olamine, N-acetylcysteine, FG-4497, AKB-4924 [29,30,31,32,33], we used for the first time Vadadustat (AKB-6548)—a novel oral PHD2 inhibitor tested in phase III clinical trials that works through the mechanism of active site iron chelation in the submicromolar range [15]. Our research has shown that Vadadustat pretreatment enhances the immunosuppressive potential of MSCs. Vadadustat significantly enhanced the suppressive effect of MSCs on PBMCs proliferation (MLR test), and this effect was partially associated with the modulation of MSCs secretome. However, the suppressive capacity of MSCs was higher in direct contact with PBMCs. This may indicate that changes in both, compounds secreted by MSCs and presented on their surface are responsible for enhancing the immunosuppressive effect of MSCs pretreated with Vadadustat. Moreover, Vadadustat significantly diminished the chemotactic properties of the MSCs secretome, as assessed by the monocyte-enriched PBMCs migration assay. It is difficult to discuss all factors whose regulation may have an effect on the immunosuppressive capacity of Vadadustat-preconditioned MSCs, but some of them are of particular importance. First of all, Vadadustat significantly decreased the expression of *IL6* and the level of secreted IL6 in relation to control MSCs. Considering that the level of IL6 can raise many thousand-fold in the course of inflammation and autoimmune diseases, we believe that MSCs preconditioning with Vadadustat may appear to be a very promising approach for the use in therapy of autoimmune diseases. Another immune-related factor that is highly regulated by Vadadustat is CXCL8 (IL8). Preconditioning with Vadadustat significantly reduces expression and secretion of CXCL8 by MSCs, as demonstrated by real-time -PCR, proteome profiler and Luminex analyzes. CXCL8 is a chemokine considered to be proinflammatory and chemotactic, especially to neutrophils. They are the most abundant group of leukocytes, constituting the indispensable line of innate immune defense against infectious diseases and their role in regulating the immune response is recently increasingly emphasized. However, neutrophils infiltration and released neutrophil extracellular traps (NETs) are also mentioned as contributing to the development of autoimmune diseases [34], especially rheumatoid arthritis (RA) [35], ANCA-associated vasculitis (AAV) and systemic lupus erythematosus (SLE) [36]. Other chemotactic factors negatively regulated in MSCs by Vadadustat were CCL7 (MCP-3) and CCL2 (MCP-1), that are both a potent monocyte-attracting chemokines. The decrease in their secretion may constitute one of the factors responsible for inhibiting monocyte-enriched PBMCs migration in the chemotaxis assay, especially when considered together with the HGF, CCL11 and CCL17. It appears that the inhibitory effect of Vadadustat preconditioning on the chemotactic properties of MSCs secretome may be therapeutically positive, as abnormal infiltration and activation of monocytes and macrophages are observed in many autoimmune diseases (reviewed in [37]). Conversely, real-time -PCR analysis showed a significant increase in the expression of another chemokine—*CCL28* after Vadadustat pretreatment. While CCL28 is responsible for the recruitment of various immune cells (which express CCR10 and CCR3) for mucosal tissue and inflammatory sites, some data indicate that it is responsible for recruiting Treg, maintaining tolerance of self antigens and preventing autoimmune diseases [38]. While there is a study demonstrating that MSCs do not express *TNF* [39], we have received its expression and increase of TNF secretion level by 237% in the secretome of Vadadustat-preconditioned MSCs (however, signal intensity indicates that this level is extremely low). It seems that such small amounts may be responsible for maintaining the MSCs immunosuppressive phenotype rather than providing wider pro-inflammatory signaling. It should also be noted that detecting such small amounts of secreted factors might give false results. Due to differences in the amount of secreted factors between MSCs populations, analyzes of pooled samples may not reflect the true trend, especially when it comes to factors secreted in small quantities. As demonstrated by Luminex, IL4 and IL10 are secreted by MSCs in very small amounts. What is more, their level decreases after Vadadustat treatment—rather than increases as the proteome profiler analysis showed—however, not statistically significantly. Therefore, the regulation of IL4 and IL10 secretion by Vadadustat pretreated MSCs, described by many authors as one of the mechanisms of MSCs immunomodulation, in this case does not seems to play a major role. It should be mentioned, however, that Vadadustat was shown to significantly reduce the secretion of another member of the IL10 family—IL24. The results obtained by real-time PCR as well as the proteome profiler showed a significant decrease in its expression and secretion. Although this cytokine is involved in the process of wound healing, the overproduction of IL24 underlies pro-inflammatory autoimmune diseases such as psoriasis, allergic contact dermatitis, atopic dermatitis, rheumatoid arthritis and inflammatory bowel disease [40,41]. Therefore, a decrease in the secretion of IL24 by MSCs as a result of Vadadustat treatment seems to be a beneficial effect when considering the use of Vadadustat preconditioned MSCs in the treatment of patients with autoimmune diseases. 

In addition to soluble factors, contact-dependent signals are also responsible for MSCs immunosuppressive activity. Our results showed that Vadadustat pretreatment caused a decrease in ICAM1 expression, while after 24 h increase in secretion of its soluble form was noted. ICAM1 is an adhesion molecule ligand for LFA-1, leukocyte integrin crucial for T cell trafficking, activation and proliferation [42]. Binding of ICAM1 with LFA-1 is involved in leukocyte endothelial transmigration. Soluble ICAM1 binding to LFA-1 was shown to inhibit lymphocyte attachment to endothelial cells (Rieckmann et al., 1995), and anti-ICAM1 or LFA-1 antibodies inhibit autoreactive T cell proliferation [43]. Therefore, the downregulation of ICAM1 expression together with increase in secretion of soluble ICAM1 after Vadadustat may constitute another mechanism for MSCs immunosuppressive activity, particularly promising in the context of inhibiting T cell autoreactivity. A more equivocal result was a Vadadustat-mediated decrease in gene expression of another cell surface molecules: PD-L1 (*PDCD1LG1*) and PD-L2 (*PDCD1L2*). Both PD-L1 and PD-L2 represent cell surface ligands for the PD-1 receptor (programmed cell death protein-1) expressed on T and B cells as an immunological checkpoint molecule. PD-1 is critical for modulating adaptive immunity by negatively regulating T-cell activation and preventing excessive or self-oriented immune responses. It is known that licensing of MSCs with proinflammatory cytokines (IFNγ, TNFα) increases the expression of PD-L1 and PD-L2 on their cell surfaces [44,45]. MSCs inhibition of T cell proliferation was reported to function through the contact dependent interaction of PD-1/PD-L1 [46,47,48]. The role of PD1 pathway in the immunomodulatory activity of MSCs is even more complex, since secretion of soluble PD-L1 and PD-L2 by BM-MSCs has also been reported [45]. We observed that the secretome from Vadadustat preconditioned MSCs inhibited the alloreactivity of PBMCs more than the secretome from control cells. Therefore, we suppose that despite the decrease in gene expression for PD-L1 and PD-L2 after pretreatment with Vadadustat, MSCs may increase the secretion of their soluble forms. However, further research is needed to define the role of Vadadustat in the by PD-L1 and PD-L2-mediated immunomodulatory function of MSCs.

In this study, we demonstrated that HIF-1 prolyl hydroxylase inhibition by Vadadustat positively affects the immunomodulatory properties of hMSCs. Preconditioning with Vadadustat has several particularly valuable features when considering the use of MSCs or MSCs secretome in the treatment of autoimmune diseases. Vadadustat, which is currently being tested for the maintenance treatment of patients with anemia secondary to chronic kidney disease in Phase III clinical studies (NCT02648347, NCT02680574, NCT04313153), aspires to become an effective tool enhancing the therapeutic activity of MSCs in the field of cell therapies.

## Supplementary Materials

The following are available online at https://www.mdpi.com/2073-4409/9/11/2396/s1, Table S1: BM-MSCs donor profile, Table S2: Flow cytometry analysis of BM-MSCs populations, Table S3: Flow cytometry results of representative BM-MSCs population, Figure S1: MTT assay.
