# Supplementary material for: Vadadustat, a HIF Prolyl Hydroxylase Inhibitor, Improves Immunomodulatory Properties of Human Mesenchymal Stromal Cells
Source: Cells. 2020 Nov 1;9(11):2396. doi: 10.3390/cells9112396 (PMC7693843; doi:10.3390/cells9112396)
Supplement: Supplementary file 1 [file cells-09-02396-s001.pdf]

cells

MDPI

Supplementary Materials

Table S1. BM-MSCs donor profile.

	Age	Sex
Donor 1	71	Male
Donor 2	68	Male
Donor 3	49	Female
Donor 4	37	Male
Donor 5	39	Male
Donor 6	39	Male
Donor 7	65	Female
Mean	52,57	
Median	49	

Table S1 shows the age and sex profile of 7 donors from which bone marrow-derived mesenchymal stromal cells (BM-MSCs) were isolated and the mean age of the donor with the median.

Table 2. Flow cytometry analysis of BM-MSCs populations.

	CD90+	CD105+	CD73+	MIX neg
Donor 1	99.7%	99.8%	99.5%	1.0%
Donor 2	99.5%	99.2%	99.6%	2.3%
Donor 3	99.1%	99.1%	99.7%	2.0%
Donor 4	99.8%	99.7%	99.4%	0.9 %
Donor 5	98.0%	98.9%	99.0%	3.2%
Donor 6	98.0%	99.7%	99.6%	0.9%
Donor 7	97.8%	99.7%	99.8%	2,0%
Mean	98.8%	99.4%	99.5%	2.4%
Median	99.1%	99.7%	99.6%	2.0%

Table S2 shows the percentage of expression of the identification markers for bone marrow-derived mesenchymal stromal cells (BM-MSCs): CD90 (Cluster of Differentiation 90), CD105 (Cluster of Differentiation 105), CD73 (Cluster of Differentiation 73) and Negative MIX (composed of CD34- Cluster of Differentiation 34; CD45 - Cluster of Differentiation 45; CD11b - Cluster of Differentiation 11b; CD19 - Cluster of Differentiation 19 and HLA-DR - Human Leukocyte Antigen DR isotype) isolated from each donor together with the mean expression value from 7 donors with the median. The analysis was performed using the BD Stemflow™ hMSC analysis kit (BD Biosciences, San Jose, CA, USA) on 7 populations of BM-MSCs at passage 4 on BD FACS Canto II using BD FACS Diva Software (BD Biosciences, San Jose, CA, USA).

Table 3. Flow cytometry results of representative BM-MSCs population.

(A) Analysis of Non-Stained BM-MSCs

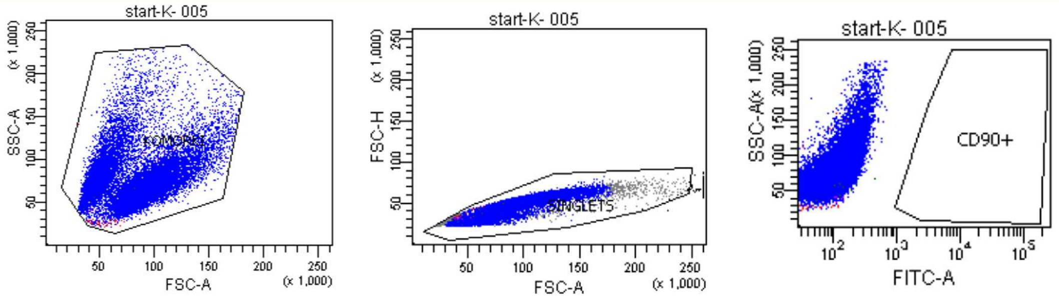

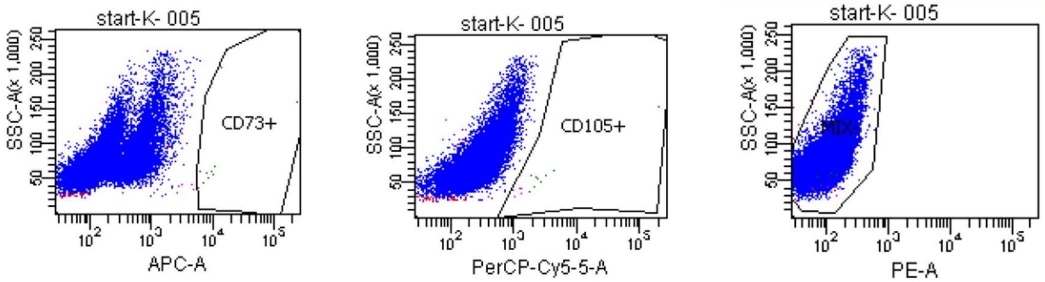

Tube: K- 005

Population	#Events	%Parent	%To
All Events	20,000	####	[illegible]
SINGLETS	19,910	99.6	[illegible]
KOMORKI	18,803	94.4	[illegible]
CD90+	0	0.0	[illegible]
MIX-	18,712	99.5	[illegible]
CD105+	16	0.1	[illegible]
CD73+	10	0.1	[illegible]
CD90+ AND MIX- AND CD105+ AND CD73+	0	0.0	[illegible]

(B) Analysis of Stained BM-MSCs

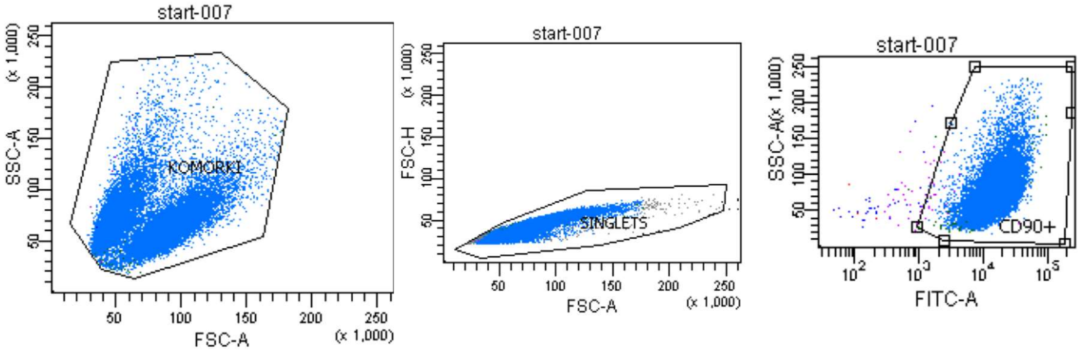

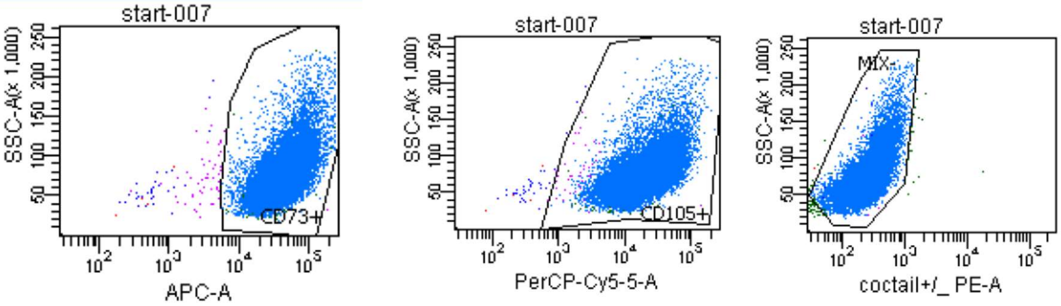

Tube: 007

Population	#Events	%Parent	%To
All Events	20,000	####	10[illegible]
SINGLETS	19,988	99.9	9[illegible]
KOMORKI	18,864	94.4	9[illegible]
CD90+	18,797	99.6	9[illegible]
MIX-	18,671	99.0	9[illegible]
CD105+	18,825	99.8	9[illegible]
CD73+	18,774	99.5	9[illegible]
CD90+ AND MIX- AND CD105+ AND CD73+	18,584	98.5	9[illegible]

Table S3 presents the results of cytometric analysis of bone marrow-derived mesenchymal stromal cells (BM-MSCs) with BD Stemflow™ hMSC Analysis Kit (BD Biosciences, San Jose, CA, USA). For the purpose of MSCs characterization, cells at passage 4 were stained with antibodies of surface markers CD105 (PerCP-Cy™5.5), CD73 (APC), CD90 (FITC) as well as negative expression markers CD45, CD34, CD11b, CD19, HLA-DR (PE). Panel A presents the results of the analysis of unstained cells: plot of the whole population (FSC-A from SSC-A), singlets (FSC-A from FSC-H), and stained for CD90, CD73, CD105 and negative cocktail with a summary table of percentage results. Panel B presents the same analysis for cells stained with anti-CD90, -CD73 and -CD105 antibodies together with a negative cocktail. The analysis was performed on BD FACS Canto II using BD FACS Diva Software (BD Biosciences, San Jose, CA, USA).

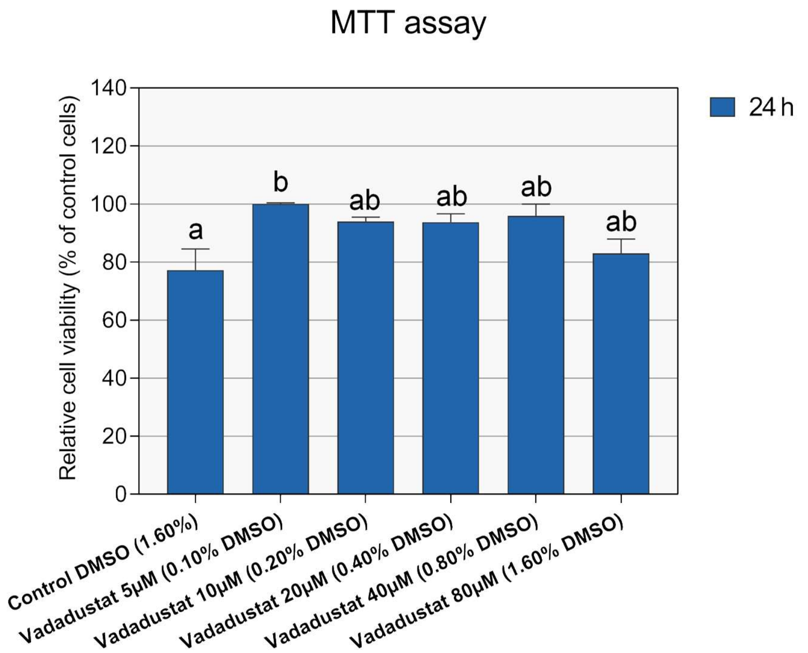

Figure S1. MTT assay. Analysis of metabolic activity of 3 bone marrow-derived mesenchymal stromal cells (BM-MSCs) populations incubated with different concentrations of Vadadustat for 24 hours. The values are presented as % of metabolic activity of control cells cultured in standard medium (without dimethyl sulfoxide -DMSO) ± SEM (standard error of the mean). Shapiro-Wilk test was used to analyze the data distribution within groups. One way ANOVA with Tukey's post test was performed to determine the statistical significance between groups at $p < 0.05$. Bars marked with the same letters (a) with (ab) as well as (b) with (ab) indicate no statistical significance between the groups, and bars marked with different letters (a) and (b) indicate statistical significant differences between the groups.
